# Precise Synaptic Efficacy Alignment Suggests Potentiation Dominated Learning

**DOI:** 10.3389/fncir.2015.00090

**Published:** 2016-01-13

**Authors:** Christoph Hartmann, Daniel C. Miner, Jochen Triesch

**Affiliations:** ^1^Department of Neuroscience, Frankfurt Institute for Advanced StudiesFrankfurt am Main, Germany; ^2^International Max Planck Research School for Neural Circuits, Max Planck Institute for Brain ResearchFrankfurt am Main, Germany

**Keywords:** SORN, STDP, synaptic normalization, synaptic scaling, homeostasis, self-organization, Kesten process, plasticity

## Abstract

Recent evidence suggests that parallel synapses from the same axonal branch onto the same dendritic branch have almost identical strength. It has been proposed that this alignment is only possible through learning rules that integrate activity over long time spans. However, learning mechanisms such as spike-timing-dependent plasticity (STDP) are commonly assumed to be temporally local. Here, we propose that the combination of temporally local STDP and a multiplicative synaptic normalization mechanism is sufficient to explain the alignment of parallel synapses. To address this issue, we introduce three increasingly complex models: First, we model the idealized interaction of STDP and synaptic normalization in a single neuron as a simple stochastic process and derive analytically that the alignment effect can be described by a so-called Kesten process. From this we can derive that synaptic efficacy alignment requires potentiation-dominated learning regimes. We verify these conditions in a single-neuron model with independent spiking activities but more realistic synapses. As expected, we only observe synaptic efficacy alignment for long-term potentiation-biased STDP. Finally, we explore how well the findings transfer to recurrent neural networks where the learning mechanisms interact with the correlated activity of the network. We find that due to the self-reinforcing correlations in recurrent circuits under STDP, alignment occurs for both long-term potentiation- and depression-biased STDP, because the learning will be potentiation dominated in both cases due to the potentiating events induced by correlated activity. This is in line with recent results demonstrating a dominance of potentiation over depression during waking and normalization during sleep. This leads us to predict that individual spine pairs will be more similar after sleep compared to after sleep deprivation. In conclusion, we show that synaptic normalization in conjunction with coordinated potentiation—in this case, from STDP in the presence of correlated pre- and post-synaptic activity—naturally leads to an alignment of parallel synapses.

## Introduction

Recent experimental evidence demonstrates that synapses from the same axonal branch to the same dendritic branch (hereafter called parallel synapses) have approximately equal strengths (Sorra and Harris, 1993; Bartol et al., 2015, and also Koester and Johnston, 2005; Kasthuri et al., 2015 for studies that did not investigate single branches). This alignment was demonstrated both in electrophysiological experiments studying synaptic efficacies in pyramidal neurons and interneurons in slices of rat LII/III somatosensory cortex (Koester and Johnston, 2005) and in EM studies on spine sizes in rat hippocampal pyramidal neurons (Bartol et al., 2015).

To explain the alignment of synaptic efficacies, Bartol et al. (2015) propose that a slow time-averaging effect is responsible for this phenomenon. Specifically, the correlation of pre- and post-synaptic firing is proposed to be independently averaged at each individual spine over long time spans by phosphorylating the calcium/calmodulin-dependent protein kinase II (CaMKII) in each spine. However, synaptic plasticity mechanisms like spike-timing-dependent plasticity (STDP) are generally thought to be temporally local (Feldman, 2012). For example, Bi and Poo (1998) induced strong changes of up to 100% within 1 min of paired 60 Hz stimulation in their seminal study on STDP.

Simultaneously, computational studies have suggested that the self-organizational interaction of additive Hebbian learning and multiplicative synaptic normalization can reproduce the experimentally observed synapse-size-fluctuations and their overall heavy-tailed distribution (Zheng et al., 2013). Synaptic normalization in this context refers to the homeostatic mechanism by which all synaptic weights are scaled by the same factor in order to balance the potentiation or depression of single weights. Experimental evidence for this can be found in the literature on “synaptic scaling” in hippocampus and neocortex (Turrigiano et al., 1998; Keck et al., 2013). Importantly, the experimental evidence suggests that this mechanism is multiplicative (Turrigiano, 2008; Keck et al., 2013), i.e., all spines grow or shrink by the same factor. While the studies around multiplicative synaptic scaling typically focus on changes on long timescales, there is also evidence for fast normalization. For example, Bourne and Harris (2011) demonstrated in EM studies in the rat hippocampus that the summed synaptic area per μm of dendrite before and after long-term potentiation protocols is roughly identical, but the area per synapse increases while the number of synapses per μm of dendrite decreases. The interaction of homeostatic mechanisms like synaptic normalization with STDP is thought to be essential for healthy circuit dynamics (Abbott and Nelson, 2000): STDP on its own tends to produce self-reinforcing potentiation since a stronger connection will make the presynaptic neuron more likely to activate the postsynaptic one. Synaptic normalization is thought to counterbalance this issue by holding the total incoming efficacy constant and only redistributing synaptic efficacies along the dendrite. We propose that a similar interaction of additive STDP with multiplicative synaptic normalization is responsible for the alignment of parallel synapses.

The underlying intuition behind this idea is as follows: When two neurons spike in short succession, the parallel synapses between them will experience the same time difference between the pre-synaptic and post-synaptic spike and therefore undergo a similar STDP-induced change, except for release failures or less prominent sources of variability. If the change is potentiating, the following multiplicative normalization will, in order to get the total synaptic weight back to its baseline, shrink both synapses yielding a smaller absolute difference compared to before the potentiating event. If, however, the change is negative, the normalization will enlarge both synapses and thereby increase the absolute difference. Therefore, we hypothesize that if potentiating events are stronger or more frequent than depressing events, this could explain the alignment observed experimentally.

We validate this intuition by first deriving a stochastic model (Model 1) for the interaction of learning and normalization. This model predicts that synaptic efficacy alignment requires a bias toward potentiation over depression during learning. This prediction is then confirmed in a simulation of a single post-synaptic neuron (Model 2) and the recurrent neural network model (Model 3) that already captures the synapse-size-fluctuations.

Please note that given that the alignment we investigate here was reported for both synaptic efficacies (Koester and Johnston, 2005) and spine sizes (Bartol et al., 2015), and given the additional extensive evidence that synaptic efficacies and spine sizes are strongly correlated (Pierce and Lewin, 1994; Schikorski and Stevens, 1997; Murthy et al., 2001), we will assume throughout the paper that the spine size can be taken as a proxy for its efficacy.

## Results

### Model 1: the interaction of learning and normalization is captured by a kesten process

The stochastic model considers a single neuron receiving multiple excitatory synaptic inputs from other neurons. Each pre-synaptic neuron can make several contacts onto the target neuron. We denote the efficacy of the *j*-th synaptic input from source neuron *i* by $X_{i}^{j}\in \text{IR}$. We describe the changes of the synaptic efficacies due to different forms of plasticity as a stochastic process. Specifically, we assume that the change of the synaptic efficacy $X_{i}^{j}$ during a short time interval can be written as a sum of two contributions from a process of Hebbian long-term plasticity and a process of synaptic normalization:

(1) $$\begin{array}{r} \Delta X_{i}^{j}\left(t\right)=P_{i}^{j}\left(t\right)+S_{i}^{j}\left(t\right)\;, \end{array}$$

where $P_{i}^{j}\left(t\right)\in \text{IR}$ is a random variable describing the change due to Hebbian long-term plasticity and $S_{i}^{j}\left(t\right)\in \text{IR}$ is a random variable describing the change due to synaptic normalization. For the former we assume that it is a product of two terms:

(2) $$\begin{array}{r} P_{i}^{j}\left(t\right)=C_{i}\left(t\right)\;F_{i}^{j}\left(t\right)\;. \end{array}$$

Here *C*_*i*_(*t*) ∈ IR is a random variable describing the *potential change* due to correlated activity of the target neuron with source neuron *i*. $F_{i}^{j}\left(t\right)$ is a binary random variable that indicates if synaptic transmission failed ($F_{i}^{j}\left(t\right)=0$) or if synaptic transmission was successful ($F_{i}^{j}\left(t\right)=1$). Therefore, a potential change of the synapse's efficacy due to correlated pre- and post-synaptic activity will only be implemented if the synapse actually transmitted the pre-synaptic spike. If transmission failed there will be no change in synaptic efficacy due to long-term plasticity. We assume that the $F_{i}^{j}$ are statistically independent. Specifically, whether the *j*-th synaptic contact from source neuron *i* fails to transmit a spike is independent of whether the *k*-th synaptic contact does so. We further assume that synaptic failure is independent of its efficacy. Later models will also consider the efficacy-dependent case. The probability distribution of *C*_*i*_ is assumed to allow for both positive and negative values corresponding to LTP and LTD, respectively. We further assume that the potential change *C*_*i*_ can be considered identical for the different parallel synaptic contacts. This is due to their close spatial proximity in Bartol et al. (2015) which suggests that almost identical currents arrive at both spines excluding effects like the distance-dependent switch from LTP to LTD reported in Froemke et al. (2005) (see Section Discussion for more details).

For the synaptic normalization we assume:

(3) $$\begin{array}{r} S_{i}^{j}\left(t\right)=\eta \left(t\right)X_{i}^{j}\left(t\right)\;, \end{array}$$

where η(*t*) ∈ IR is a random variable describing a normalization factor that is applied to multiplicatively regulate all excitatory synaptic efficacies of the target neuron. We further assume that the neuron's connectivity is in a steady state such that the average total change of synaptic efficacies due to long-term plasticity is balanced by the average change due to synaptic normalization:

(4) $$\begin{array}{rcl} \text{E}\left[\underset{i,j}{\sum }P_{i}^{j}\left(t\right)\right] & = & \text{E}\left[-\underset{i,j}{\sum }S_{i}^{j}\left(t\right)\right]=-\text{E}\left[\eta \left(t\right)\underset{i,j}{\sum }X_{i}^{j}\left(t\right)\right]\\ = & -\text{E}\left[\eta \left(t\right)T\left(t\right)\right]\;, \end{array}$$

where E[.] denotes the expected value and we have introduced $T\left(t\right)=\underset{i,j}{\sum }X_{i}^{j}\left(t\right)$ as the sum of all excitatory synaptic efficacies at a particular time. We will consider two normalization schemes to achieve this balance: *global balance* and *detailed balance*. For the *detailed balance*, we set

(5) $$\begin{array}{c} \eta (t)=-\frac{\sum_{i,j}P_{i}^{j}(t)}{\sum_{i,j}X_{i}^{j}(t)} \end{array}$$

to get instant normalization. For further analytical derivations, however, it is convenient to assume that η(*t*) is independent of *T*(*t*) such that E[η(*t*)*T*(*t*)] = E[η(*t*)]E[*T*(*t*)] = TE[η(*t*)], with T as the average total synaptic efficacy. In this *global balance* we find:

(6) $$\begin{array}{r} \text{E}\left[\eta \left(t\right)\right]=-\frac{1}{T}\text{E}\left[\underset{i,j}{\sum }P_{i}^{j}\left(t\right)\right]\;. \end{array}$$

We will later evaluate both the *detailed* and *global* balance numerically and will find that they lead to similar results for reasonable failure rates and connection densities.

To investigate the conditions under which synaptic efficacy alignment occurs, we consider without loss of generality two contacts from source neuron *i*, $X_{i}^{1}\left(t\right)$ and $X_{i}^{2}\left(t\right)$. We denote the difference of their efficacies as $D_{i}^{1,2}\left(t\right)=X_{i}^{1}\left(t\right)-X_{i}^{2}\left(t\right)$. The change of this difference during a short time interval is given by:

(7) $$\begin{array}{ll} \Delta D_{i}^{1,2}\left(t\right)= & \;\Delta X_{i}^{1}\left(t\right)-\Delta X_{i}^{2}\left(t\right)=P_{i}^{1}\left(t\right)+S_{i}^{1}\left(t\right)-P_{i}^{2}\left(t\right)-S_{i}^{2}\left(t\right)\; \end{array}$$

(8) $$\begin{array}{ll} = & \;C_{i}\left(t\right)F_{i}^{1}\left(t\right)-C_{i}\left(t\right)F_{i}^{2}\left(t\right)+\eta \left(t\right)\left(X_{i}^{1}\left(t\right)-X_{i}^{2}\left(t\right)\right)\; \end{array}$$

(9) $$\begin{array}{ll} = & \;C_{i}\left(t\right)\left(F_{i}^{1}\left(t\right)-F_{i}^{2}\left(t\right)\right)+\eta \left(t\right)D_{i}^{1,2}\left(t\right)\;.\; \end{array}$$

Thus, $D_{i}^{1,2}\left(t\right)$ obeys the following stochastic dynamics:

(10) $$\begin{array}{l} D_{i}^{1,2}\left(t+1\right)=\left(1+\eta \left(t\right)\right)D_{i}^{1,2}\left(t\right)+C_{i}\left(t\right)\left(F_{i}^{1}\left(t\right)-F_{i}^{2}\left(t\right)\right)\;. \end{array}$$

These dynamics are an instance of a so-called Kesten process (Kesten, 1973) *x*(*t* + 1) = *a*(*t*)*x*(*t*) + *b*(*t*) with *a*(*t*) = 1 + η(*t*) and $b\left(t\right)=C_{i}\left(t\right)\left(F_{i}^{1}\left(t\right)-F_{i}^{2}\left(t\right)\right)$ if *a*(*t*) and *b*(*t*) are independent.

However, it is not clear from the literature on synaptic normalization if the assumed multiplicative scaling factor (η(*t*) in *a*(*t*)) should directly depend on the potentiating and depressing events (in *b*(*t*)), or should “only” match the mean as required in Equation (4).

**Figure 1:**
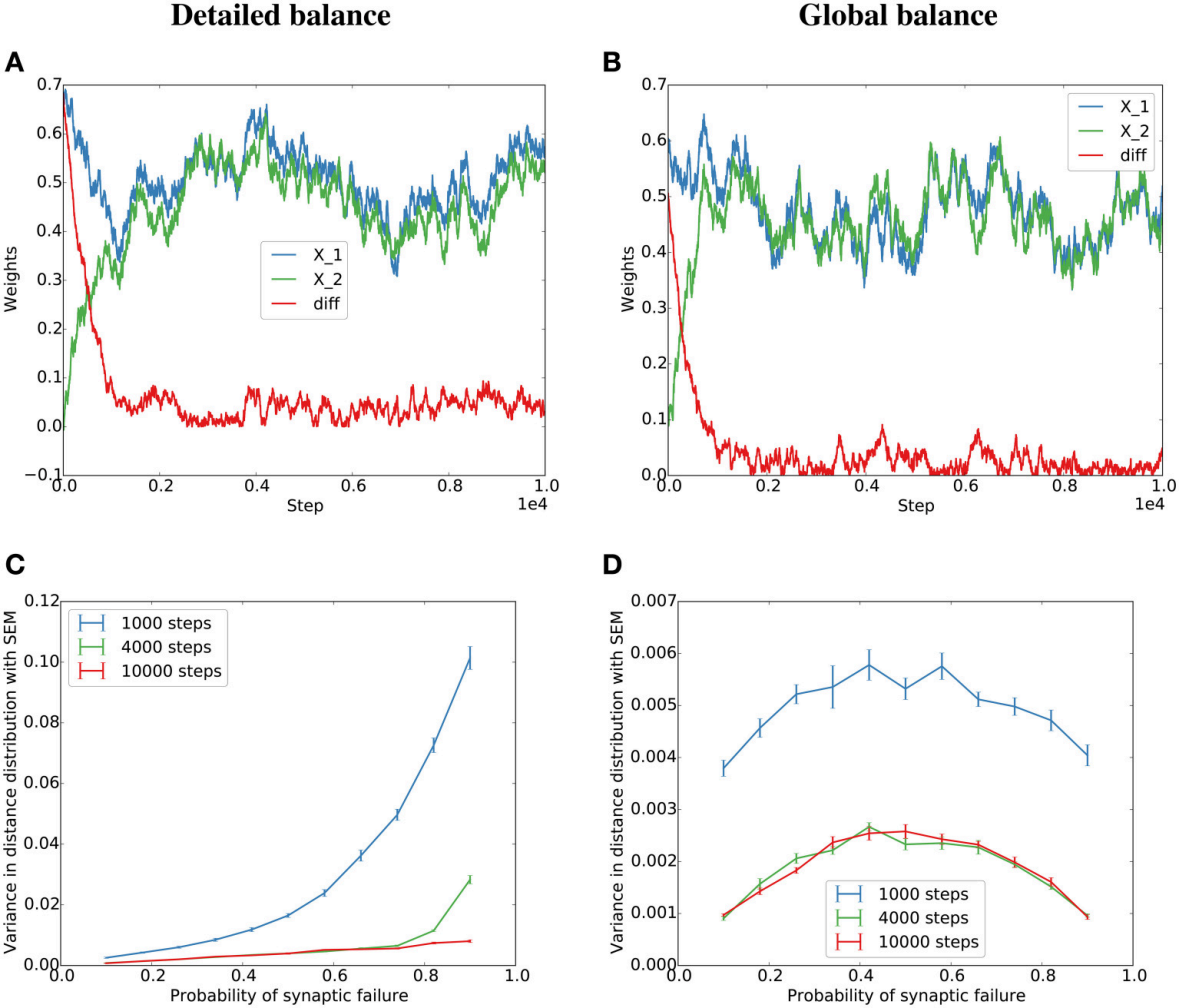
**Simulation of the Kesten process for the detailed and globally balanced η(*t*)**. **(A)** Weight traces and their absolute difference for two parallel synapses with a failure rate of 20% for the *detailed balance* condition where the normalization factor, η(*t*), is a function of the potentiating and depressing events at *t*. The potentiation was twice as strong as the depression term. **(B)** Weight traces for two parallel synapses for the *global balance* condition where η(*t*) is sampled from a distribution similar to the η(*t*) produced by the *detailed balance* in **(A)** (see Figure S1 for the distributions). The failure rate was set to 80% since for the global condition, the alignment should be the same for 20 and 80% (see main text) **(C,D)** Dependence of the variance of the final distribution on the probability of failure for the *detailed* **(C)** and *global* **(D)** condition.

To test both cases, we simulate a *detailed balance* condition where η(*t*) balances the total potentiation and depression at each time step (Equation 5), and a *global balance* condition where η(*t*) is drawn independently from a distribution matched to the experimental distribution of η(*t*) in the *detailed balance* condition (see Figure S1 for the distributions and Methods for more details). The simulations lead to similar behavior for low failure rates or many pre-synaptic partners (Figure 1). This is because for many partners or low failure rates, many individual events in *b*(*t*) accumulate in η(*t*) and thereby minimize the dependence of η(*t*) on individual potentiating or depressing events.

In summary, the interaction of STDP and synaptic normalization can be approximated by a Kesten process. This can now be used to derive conditions for synaptic efficacy alignment.

### Potentiation must dominate for the kesten process to converge

The qualitative behavior of the Kesten process depends on the distribution of *a*(*t*). The Kesten process has a limiting distribution if and only if E[ln *a*(*t*)] < 0 (Sornette and Cont, 1997; Statman et al., 2014). In terms of the Kesten process derived before, a limiting distribution would entail convergence of the difference $D_{i}^{1,2}$ over time to a fixed distribution, i.e., alignment up to a certain precision. If, however, there would not be a limiting distribution, the absolute difference $D_{i}^{1,2}$ would keep increasing indefinitely. By applying the condition for a limiting distribution to our model, we can see that the condition for the existence of a stable limiting distribution in our case is E[ln(1 + η(*t*))] < 0. Since the absolute value of η(*t*) can be assumed to be very small (recall that it is of the order of change in synaptic efficacy over total synaptic efficacy), we use the first-order Taylor expansion ln(1 + η(*t*)) ≈ η(*t*) to find the condition:

(11) $$\begin{array}{r} 0>\text{E}\left[\eta \left(t\right)\right]=-\frac{1}{T}\text{E}\left[\underset{i,j}{\sum }P_{i}^{j}\left(t\right)\right]\;. \end{array}$$

**Figure 2:**
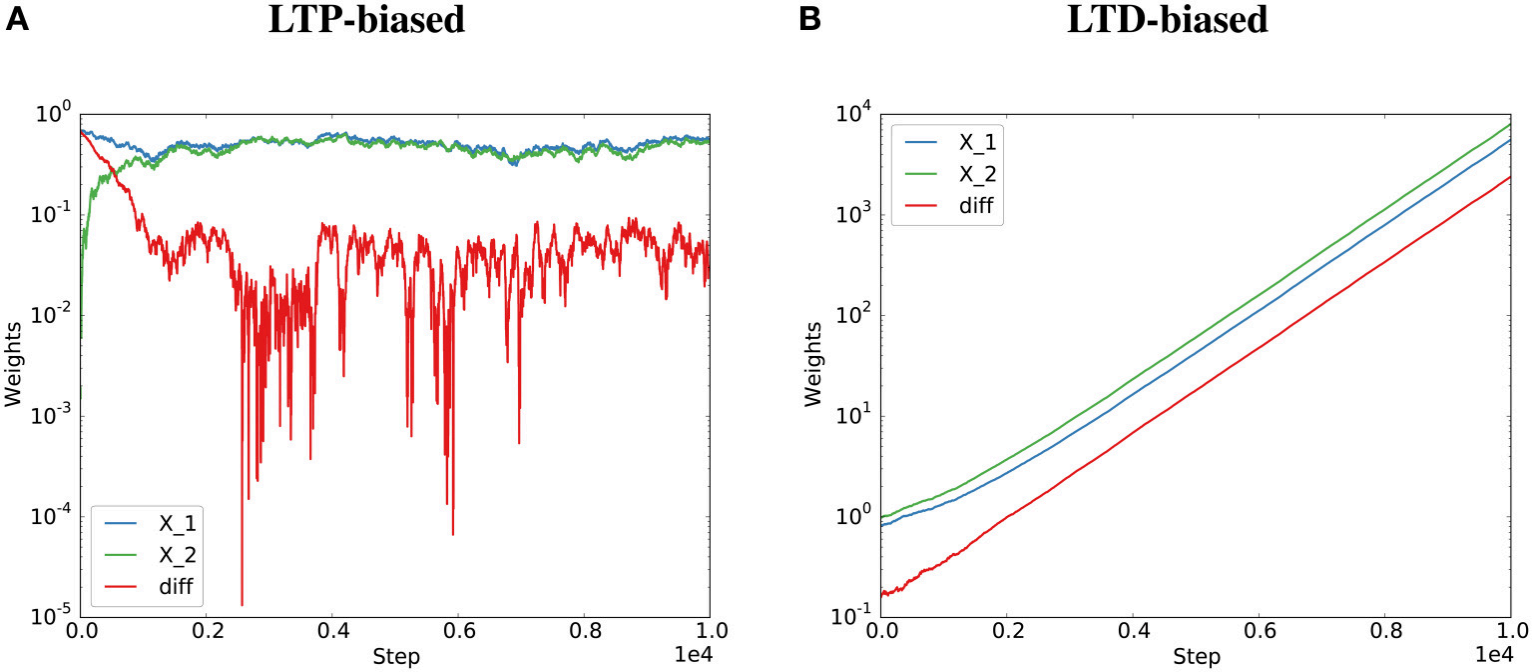
**Potentiation must dominate for the Kesten process to converge**. The Kesten process was instantiated with a synaptic failure probability of 20% for each synapse and potentiation twice as strong as depression **(A)** or half as strong as depression **(B)**. The normalization constant, η(*t*), was at each step set to the total potentiation and depression divided by the total spine sizes to get the required steady state (*detailed balance* condition, see Figure 1). Parallel synapses align for the LTP-biased condition (**A**, same data as Figure 1A) and diverge for the LTD-biased condition **(B)**. The final average distances for 100 spine pairs was 0.03 for the LTP-biased case and 5747 for the LTD-biased simulation. When simulating the process for ten times as many steps, the final average distance becomes around 0.035 for the LTP-biased case and around 10^16^ for the LTD-biased simulation. Please note the different scales for the two plots: the weights become much larger in the LTD-biased simulation. Therefore, the comparatively small fluctuations due to synaptic failure that are still visible at the beginning of **(B)** quickly become invisible with more simulation steps.

Therefore, the behavior depends on the expected total change in synaptic efficacy due to Hebbian plasticity. If $\text{E}\left[\underset{i,j}{\sum }P_{i}^{j}\left(t\right)\right]<0$, i.e., synaptic efficacy changes due to Hebbian plasticity are dominated by depression, then E[η(*t*)] > 0 and the strength of the two synapses is not guaranteed to converge to a limiting distribution. The synapses are not guaranteed to align their efficacies. If $\text{E}\left[\underset{i,j}{\sum }P_{i}^{j}\left(t\right)\right]>0$, i.e., synaptic efficacy changes due to Hebbian plasticity are dominated by potentiation, then E[η(*t*)] < 0 and the strength of the two synapses is guaranteed to converge to a limiting distribution. The two synapses will align their efficacies. This was validated by artificially biasing the simulations of the Kesten process toward “long-term potentiation” (LTP) or “long-term depression” (LTD) by drawing the *C*_*i*_ terms from distributions biased toward positive or negative values (see Figure 2 and Methods for details).

In the case that potentiation dominates, the variance of the limiting distribution and therefore the precision of synaptic efficacy alignment will depend on the variance of $b\left(t\right)=C_{i}\left(t\right)\left(F_{i}^{1}\left(t\right)-F_{i}^{2}\left(t\right)\right)$. The factor *C*_*i*_(*t*) indicates that the imprecision of alignment, i.e., the variation from perfect alignment, scales with the overall amplitude of fluctuations of synaptic efficacies due to Hebbian plasticity. The second term reflects the role of synaptic failures. For the sake of simplicity, let us assume that synaptic failures occur independently with a probability *f* that is independent of the synapse's efficacy. The difference of the *F*_*i*_ then takes the value 0 with probability *f*^2^ + (1 − *f*)^2^ and the values ±1 with probability *f*(1 − *f*). The variance of $F_{i}^{1}\left(t\right)-F_{i}^{2}\left(t\right)$ is then given by 2(*f* − *f*^2^). Thus, a small synaptic failure rate leads to a precise alignment of the synapses. The variance is biggest when *f* = 1∕2, i.e., when the *F*_*i*_ have their maximum entropy of 1 bit. In this case their difference also assumes its maximum entropy of 3∕2 bits and synaptic efficacy alignment is expected to be least precise.

These results are confirmed in a simulation of a corresponding Kesten process with independent η(*t*) as described in the previous section (Figures 1B,D). However, when η(*t*) depends on the potentiating and depressing events at *t* (*detailed balance*), the variance increases with failure probability (Figure 1C). In the case of high failure probabilities, there are only very few potentiating or depressing events at each time step, which leads to a high correlation between *a*(*t*) and *b*(*t*) of the model dynamics and thereby to a violation of the assumptions of the derivations in this section. Nevertheless, the key result that parallel synapses align in potentiation-dominated regimes holds also for the *detailed balance* condition at reasonable failure probabilities (Figure 2).

### Model 2: potentiation-dominated alignment is verified for model synapses

Next, we test this idea, that synapses align in a potentiation-dominated regime through the interaction with synaptic normalization, in more realistic models. We first test this prediction by simulating a single model neuron with 1000 pairs of incoming synapses for a total of 2000 synapses. Each pair of synapses receives spikes from an independent Poisson spike source at a rate of 1 Hz. The post-synaptic spike train is an additional independent Poisson spike source with a rate of 1 Hz. Each synapse is subject to exponential spike-timing-dependent plasticity (STDP; Feldman, 2012). This is a process by which synaptic weights are modified by the pairing of pre- and post-synaptic spikes according to their relative timing within two exponential windows, one for depression (the post-before-pre case) and one for potentiation (the pre-before-post case). This provides the additive component of the Kesten-equivalent process.

The totality of synapses are subject to synaptic normalization (SN; Turrigiano, 2008). Since this is thought to be a multiplicative process, we implement it by multiplicatively renormalizing all weights after plasticity events to a constant total incoming weight (see Sections Introduction and Methods for details). This provides the multiplicative component of the Kesten-equivalent process. It should be noted that this is not a “pure” Kesten process, as the possible values for the synaptic weights are necessarily truncated below zero to conform with Dale's law.

Synaptic failure is modeled as random and independent with a probability of 0.2 (Hardingham and Larkman, 1998). Initial weights are randomly selected from a uniform distribution between 0 and 5 mV. The independence of the post-synaptic spike train (as opposed to simply modeling the post-synaptic neuron as a leaky integrate-and-fire neuron) eliminates any correlations between pre- and post-synaptic spike trains, which tend to bias STDP toward potentiation (see next section). This then allows us to simply tune the STDP windows to control potentiation or depression dominance.

**Figure 3:**
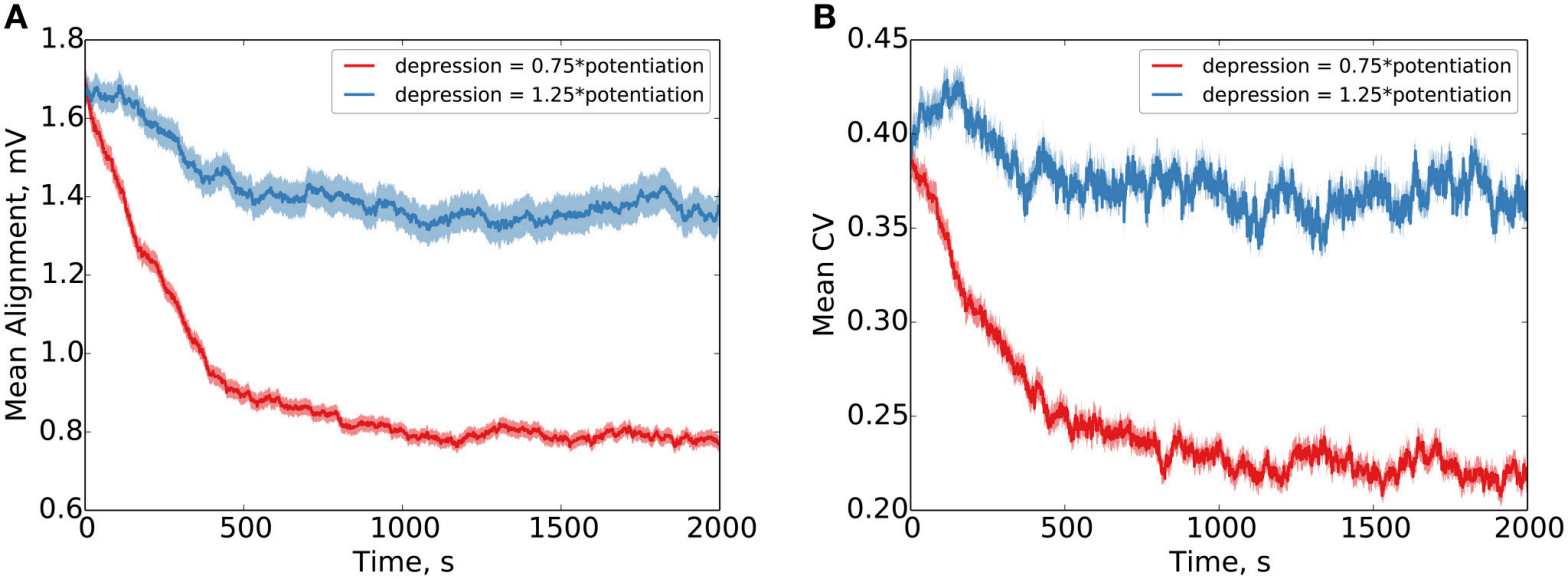
**Parallel synapses on a single postsynaptic neuron align with synaptic normalization and LTP-biased STDP**. Behavior under LTP-biased STDP (depression 0.75 × as strong as potentiation) and LTD-biased STDP (depression 1.25 × as strong as potentiation) is examined. Under the LTP-biased condition, the mean absolute difference in efficacy between parallel synapses decreases in time before reaching a fixed value **(A)**. Similarly, under these conditions, the mean degree of alignment measured by the coefficient of variation (CV, $\frac{\sigma }{\mu }$) between parallel synapses also decreases in time before reaching a fixed value **(B)**. Conversely, under LTD-biased STDP, both the mean absolute difference in efficacy and the mean CV between parallel synapses fail to readily converge. Red shading shows the standard error of the mean over 1000 weight pairs.

Our results clearly demonstrate precise alignment of synaptic efficacies in the case that the depressing window is given 0.75 × the amplitude of the potentiating window in the STDP (Figure 3). In the case that the depressing window is given 1.25 × the amplitude of the potentiating window, failure to precisely align is demonstrated (the synaptic normalization in this case, compared to the Kesten process, provides bounds on the maximum and minimum possible weights, limiting the degree of divergence). Our simulations use additive STDP, but qualitatively equivalent results are observed for multiplicative STDP as well (see Figure S2).

### Model 3: recurent interactions can lead to potentiation-dominated regimes and alignment

In order to transfer these results to recurrent neural circuits, we decided to model synaptic efficacy alignment in the self-organizing recurrent neural network model (SORN, Lazar et al., 2009). This model consists of an excitatory and inhibitory population of synchronously updated threshold units. They and their connections are acted upon by four key plasticity mechanisms: spike-timing-dependent plasticity (STDP, Feldman, 2012) shapes the connections within the excitatory population and from the inhibitory population to the excitatory population. New synapses are randomly inserted by structural plasticity. All recurrent excitatory synapses are balanced by synaptic normalization (SN, Bourne and Harris, 2011) and the firing thresholds of each unit are homeostatically regulated by intrinsic plasticity (IP; Desai et al., 1999). We chose to use this specific model for a recurrent circuit because it has been shown to nicely capture fluctuations of individual spine sizes and their overall log-normal-like distribution (Zheng et al., 2013). The model was modified to have two parallel synapses between connected excitatory neurons, a possibility to bias the STDP rule toward potentiation or depression and independent synaptic failure. All other mechanisms and parameters were kept identical to Zheng et al. (2013) (see Section Methods for details). The main results reported here also hold for weight-dependent synaptic failure (see Figure S6).

**Figure 4:**
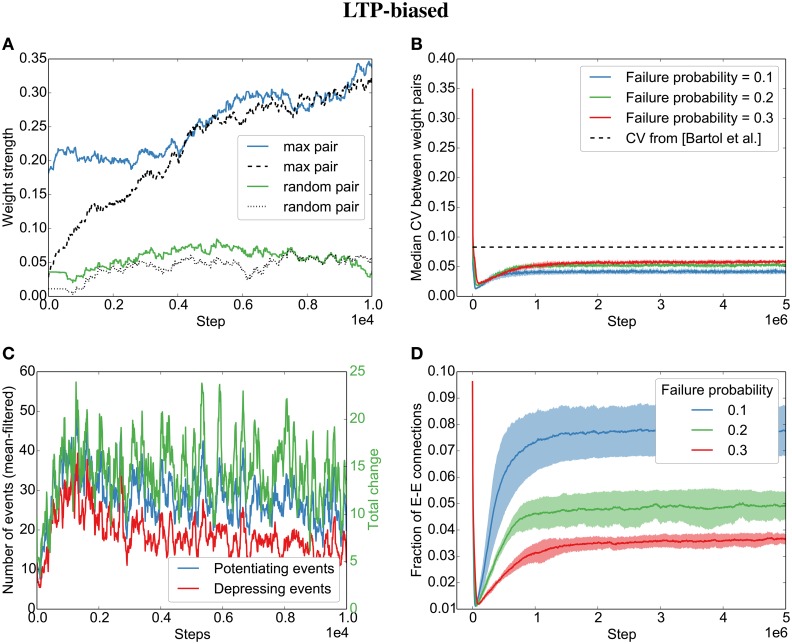
**Parallel synapses align in recurrent circuits with LTP-biased STDP**. The SORN model was simulated with 20% failure probability for each synapse and potentiation 1.25 times as strong as depression. **(A)** Parallel synapses align. The pair with the maximal final mean weight (blue and dashed) and a randomly selected pair (green and dotted) are plotted for the beginning of the simulation. **(B)** The divergence measured by the coefficient of variation (CV) drops below the experimentally reported values for different failure probabilities. **(C)** STDP dynamics at the beginning of the simulation. There are fewer depressing events (red line) than potentiating events (blue line) and the total weight change due to STDP is positive (green line). **(D)** The overall fraction of excitatory-to-excitatory connections converges and follows a qualitatively similarly fast decay and slow increase as reported in the original paper (Zheng et al., 2013) for different failure probabilities. Shaded areas are standard deviations over five independent simulations.

As for Model 2, with independent Poisson spike trains in the previous section, we observe an alignment of synapses when STDP is biased toward long-term potentiation (LTP) (Figures 4A,B). This is due to an average positive weight change (Figure 4C, green line).

**Figure 5:**
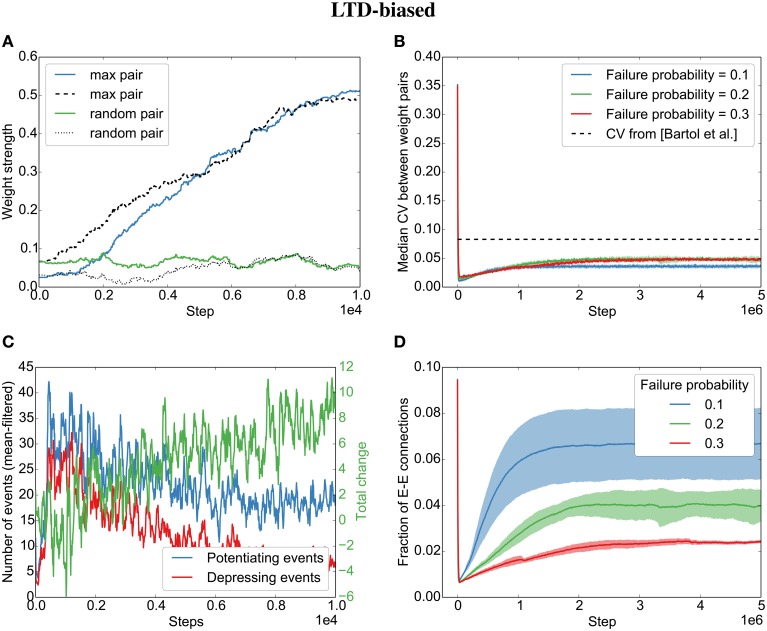
**Parallel synapses align in recurrent circuits with LTD-biased STDP**. The SORN model was simulated with 20% failure probability for each synapse and depression 1.25 times as strong as potentiation. **(A)** Parallel synapses align. The pair with the maximal final mean weight (blue and dashed) and a randomly selected pair (green and dotted) are plotted for the beginning of the simulation. **(B)** The CV drops below the experimentally reported values for different failure probabilities. **(C)** STDP dynamics at the beginning of the simulation. After a short initial period, there are fewer depressing events (red line) than potentiating events (blue line) and the total weight change due to STDP becomes positive positive (green line). **(D)** The overall fraction of excitatory-to-excitatory connections converges and follows a qualitatively similarly fast decay and slow increase as reported in the original paper (Zheng et al., 2013) for different failure probabilities. Shaded areas are standard deviations over five independent simulations.

However, contrary to what we found for the independent spike trains, we observe that even when biasing the STDP rule toward LTD, the parallel synapses will eventually align (Figures 5A,B). This is due to plasticity shaping network dynamics: The dominant depression will almost instantaneously prune most synapses between neurons that fire in post-before-pre order so that barely any connections are left that might be subject to depression (see the quickly decreasing number of depressing events in Figure 5C). However, those synapses that are aligned to the sequential firing of two neurons are not affected by this and will be potentiated, making it even more likely for the two neurons to fire in succession and therefore to receive more potentiation (see development toward net positive change (green line) in Figure 5C). This eventually leads to the emergence of synfire-like firing patterns (Zheng and Triesch, 2014). This self-reinforcing correlation of firing then leads to the alignment of the remaining synapses.

Interestingly, both the LTP-biased simulation and the LTD-biased simulation display the same three dynamic regimes as in the original paper (Zheng et al., 2013): After an initial fast decay of excitatory connections, they slowly increase again until the pruning by STDP reaches an equilibrium with the creation of new synapses by the structural plasticity. Despite the different speeds of the growth phases in the LTP- and LTD-biased simulations, they both reach a similar equilibrium point (connection fraction of about 5% for a failure probability of 0.2) and a similar median divergence measured by the coefficient of variation (CV,$\frac{\sigma }{\mu }$) between weight pairs (≈ 0.05). So while biased STDP seems to have an effect for the initially random connectivity matrix, the bias on the STDP windows does not seem to matter for the final connectivity statistics due to the recurrent dynamics of the model.

While the bias barely affects the final result, the probability of synaptic failure does have an effect. The lower the probability of failure, the higher the final connection fraction (Figures 4D,5D). Nevertheless, the coefficient of variation stays below the reported value (0.083 in Bartol et al., 2015) for the tested probabilities (0.1 − 0.3).

**Figure 6:**
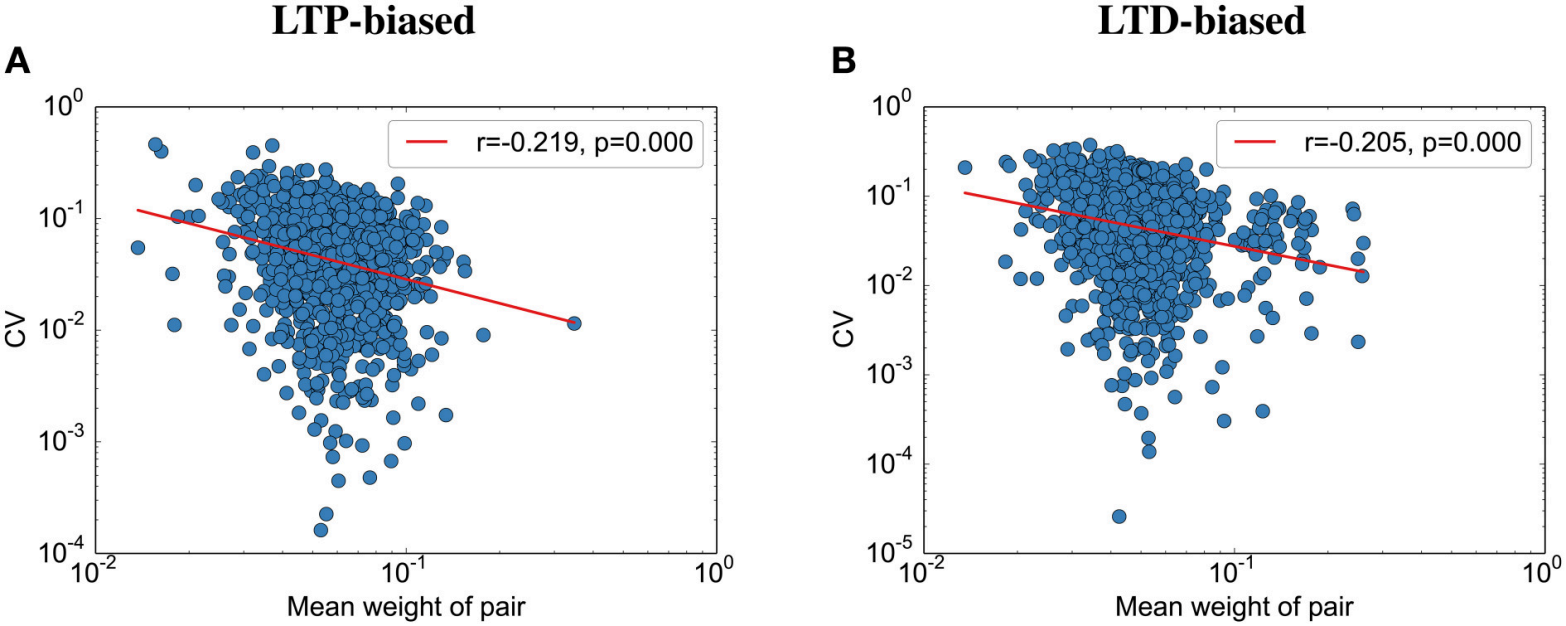
**The coefficient of variation depends on the spine size**. **(A)** CV vs. simulated spine size for the LTP-biased simulation in Figure 4. The red line is a power-law fit, *p* gives the significance of the correlation, *r* the correlation coefficient. **(B)** CV vs. simulated spine size for the LTD-biased simulation in Figure 5.

Contrary to the results reported by Bartol et al. (2015), we find that the final weight and the CV are weakly correlated (Figure 6): the stronger the weight, the lower the CV to its partner. This seems to be due to the nature of fluctuations toward the end of the simulation: All synapses are almost aligned, but due to the unreliable individual synapses, some variability remains. Because we assumed here that the probability of failure is independent of the synaptic size, this standard deviation between weight pairs (σ) should not vary with size. Following this logic, larger weight pairs (with a larger mean weight μ) should have a lower CV than smaller weight pairs since the CV is defined as $\frac{\sigma }{\mu }$. Please note that while there is data that release probability is dependent on synaptic size, this would only make this effect more prominent since the experimental data usually show that stronger weights have a lower probability of failure (Koester and Johnston, 2005), making their standard deviation smaller than the standard deviation between small synapse pairs (assuming detailed balance, as used in this model).

## Discussion

In conclusion, we find that synaptic normalization in conjunction with a potentiation-dominated activity regime leads to the alignment of parallel synapses. The dominance of potentiation can either be achieved with LTP-biased STDP or the correlation-induced potentiation dominance in recurrent circuits. This is in line with recent findings suggesting a dominance of potentiation over depression during waking and down-normalization during sleep (Tononi and Cirelli, 2014). However, the separation of learning and normalization is at odds with the simplified instantaneous synaptic normalization we used so far. As further explained below, we also validated our results for synaptic normalization on slower timescales. This leads us to predict that individual spine pairs will be more similar after sleep compared to sleep deprivation.

While some forms of synaptic normalization redistribute efficacies immediately with LTP or LTD (Bourne and Harris, 2011), others seem to work on a slower timescale (Turrigiano, 2008; Tononi and Cirelli, 2014). This is especially important with regards to our prediction that the variability is smaller after sleep: If normalization takes place mainly during sleep, our results also have to hold for normalization on longer timescales. This is true for both our derivation and the simulations: the derivation of the Kesten process does not require this assumption for convergence and we found that normalizing on a slower time scale does not affect the key findings presented here (Figures S2, S5).

A further assumption of the analytical model and subsequent simulations is that the normalization is acting in a multiplicative way: all weights are scaled by a common factor instead of, for example, adding or subtracting a small common value from all weights. While the experimental evidence suggests that synaptic scaling is indeed multiplicative (Turrigiano et al., 1998; Keck et al., 2013), the studies investigating this only focused on synaptic scaling at slow timescales on the order of days. Also, the experimental evidence for multiplicative scaling usually only focuses on a scaling of the cumulative distribution of efficacies as opposed to the scaling of individual efficacies due to experimental challenges. This scaling of the distribution might also be explained by other processes than a scaling of each individual efficacy (Statman et al., 2014). Generally speaking, multiplicative normalization induces less competition between synaptic weights compared to subtractive normalization, where weights tend to either be pruned away or converge to high values (Miller and MacKay, 1994). This seems to be at odds with the log-normal-like distribution of synapse strength in neocortex (Buzsáki and Mizuseki, 2014). Nevertheless, more research is needed to determine whether synaptic normalization at faster timescales (Bourne and Harris, 2011) is also multiplicative. With regards to our own experiment, we have run a single neuron simulation using such subtractive normalization (in which case, it should be noted that the system can no longer be modeled as a Kesten process), and noted that alignment is weak or absent in this case (see Figure S4). This suggests that where synaptic efficacy alignment is strongly present in the brain, scaling is more likely to be multiplicative than additive. Along similar lines, we considered the effects of weight-dependent synaptic failure on the single neuron model, in which case alignment is present but weakened, and sensitive to the failure function (see Figure S4), and in the SORN model (Figure S6) with qualitatively similar results to weight-independent failure. Please note that this modification would compromise the independence-assumption of the *C*_*i*_ term in the Kesten process. Finally, we also tested multiplicative STDP for the single-neuron model (see Figure S3), in which case the alignment conditions are roughly maintained, but the process is again no longer a Kesten process.

Another assumption of our model is the equivalence of currents arriving at parallel synapses and thereby the equivalence of induced plasticity events when both synapses transmit their signal. This seems to be at odds with studies like Froemke et al. (2005) that show that the form of plasticity can change depending on the distance from the soma. To get a better grasp on this, it is useful to review the literature on synaptic alignment: Koester and Johnston (2005) first showed with simultaneous *Ca*^2+^ imaging and dual whole-cell recordings that synaptic efficacy pairs within or between pyramidal neurons and interneurons in slices of rat LII/III somatosensory cortex are correlated [see Figure 4c of Koester and Johnston (2005)]. However, the synaptic pairs they report were not on the same pre- and post-synaptic branch but potentially on different branches. The different branches and the resulting high distance between the pairs (up to 300μ*m*) suggests that the pre- and post-synaptic currents arriving at the synapses differ in amplitude and timing, which might explain the remaining variability in the data by, for example, distance-depenent STDP (Froemke et al., 2005). A complementary electron microscopy (EM) study was recently performed in mouse somatosensory cortex (Kasthuri et al., 2015). This study, too, found that synaptic pairs with the same pre- and postsynaptic neuron are more similar than synapses with different targets or sources. However, this study also did not distinguish individual axonal and dendritic segments. This gap was closed by Bartol et al. (2015), updating an old and less precise study from the same group (Sorra and Harris, 1993). In their EM study, the alignment of parallel synapses, i.e., synapses from the same axonal branch onto the same dendritic branch, between rat hippocampal pyramidal neurons was found to be highly precise. Furthermore, parallel synapses are much more precisely aligned than synapses from the same axon onto different dendritic branches, presumably due to different activation histories. While the distance between them did not affect the alignment, the distance was in general very small due to the same-branch constraint (less then 5μ*m*) excluding effects of distance-dependent differences in STDP (Froemke et al., 2005). Since we focus on modeling the results of Bartol et al. (2015), it is reasonable to assume that the plasticity events at parallel synapses are equivalent if both synapses transmit.

Finally, as mentioned already by Bartol et al. (2015), there are more possible disturbances to the synaptic efficacy alignment than just synaptic failure. Here, we only considered synaptic failure because it seems to be the strongest source of variability for the alignment process and modeling all sources of variability would have made the model too complicated. However, all of the circuit simulations presented here lead to CVs below the experimentally reported value (Figures 4, 5, Figure S5). This indicates that additional disturbing processes could be included while still reaching the reported CV with our proposed interaction of correlation-based learning with synaptic normalization.

Interestingly, while we were able to replicate most of the results on synaptic efficacy alignment, we differ from Bartol et al. (2015) in that with our mechanisms the CV between weight pairs shrinks with the mean weight. As explained in the results, this is not due to our assumption that all weights have similar failure probabilities. We therefore predict, in addition to the lower overall variability after sleep, a lower variability for larger weights. Bartol et al. (2015) might not have found this effect due to their small sample size.

While we are, to the best of our knowledge, the first to show that the interaction of STDP and synaptic normalization can be described by a Kesten process, we are not the first to use the Kesten process to describe spine size fluctuations: Statman et al. (2014) used a Kesten process to stochastically describe spine size fluctuations observed experimentally. Interestingly, similar spine size fluctuations were also explained by the SORN model used here that depends on the interaction between STDP and homeostatic mechanisms to capture the spine size fluctuations (Zheng et al., 2013). The analytical result presented here that the interaction of STDP and synaptic normalization can effectively be described as a Kesten process suggests that (Statman et al., 2014) and (Zheng et al., 2013) are just two sides of the same coin.

Taken together, we were able to show both analytically and in simulations that the precise synaptic efficacy alignment observed experimentally could be a direct implication of time-local STDP with synaptic normalization in recurrent circuits. Despite the simplicity of the models presented here, we can make the specific predictions that the alignment precision is correlated to the spine size and that the alignment should be better after a good night's sleep than after a day at the lab.

## Methods

### Kesten process simulation

For the Kesten process simulation, we iterate through different instantiations of the process in the following way: First, the synaptic pairs *X*^1^(0) and *X*^2^(0) are independently initialized for *N* = 100 pre-synaptic neurons in the interval [0, 1]. Next, we update *X*^*j*^ at each time step *t* the following way:

- A correlation term *C*(*t*) is drawn at each step uniformly in the interval [−0.005, +0.005] for each pre-synaptic neuron. We then multiply all positive entries of *C*(*t*) with a LTP bias.
- The failure variables *F*^1^(*t*) and *F*^2^(*t*) are drawn randomly for each synapse according to their failure probability
- *P*^1^(*t*) and *P*^2^(*t*) are computed as *P*^*j*^(*t*) = *C*(*t*) × *F*^*j*^(*t*)
- For the *detailed balance* condition, η(*t*) is then set to $-\frac{\overset{N}{\underset{i=0}{\sum }}P_{i}^{1}\left(t\right)+P_{i}^{2}\left(t\right)}{\overset{N}{\underset{i=0}{\sum }}X_{i}^{1}\left(t\right)+X_{i}^{2}\left(t\right)}$. For the *global balance* condition, η(*t*) is drawn from the experimentally determined *detailed balance* distribution for a LTP bias of 2 and failure probability of 20%: η(*t*) ~ *normal*(μ = −0.002, σ = 0.001)
- Finally, we set *X*^*j*^(*t* + 1) = *X*^*j*^(*t*) + Δ*X*^*j*^(*t*) with Δ*X*^*j*^(*t*) = *P*^*j*^(*t*) + η(*t*)*X*^*j*^(*t*)

The simulation code is available as an ipython notebook on github.com/chrhartm/SORN.

### Single-neuron model

Each synapse in our model is endowed with exponential spike-timing-dependent plasticity (STDP) (Bi and Poo, 1998; Feldman, 2012). This defines the weight change to a synapse caused by a pair of pre- and post-synaptic spikes as follows:

(12) $$\begin{array}{l} \Delta w_{j}=\overset{N_{m}}{\underset{m=1}{\sum }}\overset{N_{n}}{\underset{n=1}{\sum }}W\left(t^{n}-t_{j}^{m}\right) \end{array}$$

(13) $$W(x)=\{\begin{array}{l} A_{+}\exp\left(-x/\tau_{+}\right),\;\;\;\;\;\;x>0\\ A_{-}\exp\left(x/\tau_{-}\right),\;\;\;\;\;\;\;\;\;x<0 \end{array}$$

Here, *W* defines the weight change window, *j* indexes the synapse, and *m* and *n* index pre- and post-synaptic spikes respectively (and their corresponding *N*s are the total number of spikes in an arbitrary period sufficiently long for the window given by *W* to have decayed to near zero). *A*_+_ and *A*_−_ are the maximal amplitudes of the weight changes, and τ_+_ and τ_−_ are the time constants of the decay windows. Values are set to approximate experimental data; in particular, round numbers were chosen that roughly approximate data in Bi and Poo (1998), with τ_+_ = τ_−_ = 20 *ms*, *A*_+_ = 0.001, and *A*_−_ = [0.00075, 0.00125], depending on our chosen balance condition. We use the “nearest neighbor” convention in order to efficiently implement this online, in which only the closest pairs of pre- and post-synaptic spikes are taken.

Additionally, synapses are endowed with stochastic failure, in which a pre-synaptic spike has no effect on the synapse. The failure rate is set to 0.2, in accordance with measurements from rodent cortical cells at body temperature (Hardingham and Larkman, 1998).

We desired a synaptic normalization model that would be simple to implement and could provide a variable timesecale. To this end, we perform the normalization operation as follows:

(14) $$\begin{array}{l} w_{j}\leftarrow \;w_{j}\left(1+\eta_{\text{SN}}\left(\frac{W_{\text{total}}}{\sum_{k}^{N}w_{k}}-1\right)\right) \end{array}$$

Here, *w*_*j*_ is the weight for an incoming synapse to the test neuron, *w*_*k*_ are the weights of all the individual synapses, *W*_*total*_ is the target total input for the neuron, and η_*SN*_ is a rate variable which determines the timescale of the normalization. *W*_*total*_ is calculated before the simulation run as *W*_*total*_ = [synapses per set (2)] × [numer of sets (1000)] × [maximum initial weight (5 mV)] × [0.5] = 5*V*. To simplify the model, we use instantaneous normalization, i.e., η_*SN*_ = 1.0. Testing has shown that this does not have a qualitative effect on the end result of the simulation (see Figure S3).

The simulation is implemented in the BRIAN simulator (Goodman and Brette, 2008). Simulation timestep is 0.1 ms, while data is sampled at intervals of 10 ms. The corresponding code is available on http://fias.uni-frankfurt.de/~miner/.

### SORN model

The recurrent network model is based on the self-organizing recurrent neural network model (SORN, Lazar et al., 2009; Zheng et al., 2013). For this study, we keep all parameters identical to a recent study on explaining weight fluctuations and log-normal weight distributions observed experimentally (Zheng et al., 2013). The model is modified to include parallel synapses, biased STDP and synaptic failure as follows. The corresponding code is available on github.com/chrhartm/SORN.

With the paired synapses, the state update equations take the following form:

(15) $$\begin{array}{l} x(t+1)=\Theta (W_{1}^{EE}(t)x(t)+W_{2}^{EE}(t)x(t)-W^{EI}(t)y(t)\\ \;-T^{E}(t)+\xi^{E}(t)) \end{array}$$

(16) $$y(t+1)=\Theta (W^{IE}x(t+1)-T^{I}+\xi^{I}(t))$$

where ***x*** is the vector of excitatory activations for *N*^*E*^ = 200 neurons and ***y*** the inhibitory activations for *N*^*I*^ = 0.2 × *N*^*E*^ = 40 neurons. **T**^*E*^(*t*) and **T**^*I*^ are their respective thresholds. They are initialized uniformly in the interval [0, 1] for the excitatory thresholds and [0, 0.5] for the inhibitory ones. ξ stands for Gaussian noise with μ = 0 and σ^2^ = 0.05. The Heaviside step function Θ(·) enforces a binary representation at each step by setting ***x***_*i*_ = 1 if the argument is greater than zero. **W**^*EI*^(*t*) is the connectivity matrix from the inhibitory to the excitatory units with an initial connection fraction of *p*^*EE*^ = 0.2. The connections from the excitatory to the inhibitory neurons, **W**^*IE*^, are densely populated. ${\text{W}}_{1}^{EE}$ and ${\text{W}}_{2}^{EE}$ stand for the parallel excitatory connectivity matrices. Initially these are set to have the same connections pairs but different individual strength in the interval (0, 1) with a connection probability of *p*^*EE*^ = 0.1.

The STDP rule acts on both ${\text{W}}_{1}^{EE}$ and ${\text{W}}_{2}^{EE}$ independently. The positive and negative parts are scaled according to β to make the potentiation part β times as strong as the depression $\left(\frac{\delta_{pot}}{\delta_{dep}}=\frac{\eta_{STDP}*\beta^{\frac{1}{2}}}{\eta_{STDP}*\beta^{-\frac{1}{2}}}=\beta \right)$:

(17) $$\begin{array}{l} \Delta {\text{W}}^{EE}\left(t+1\right)=\eta_{STDP}\left(\beta^{\frac{1}{2}}\text{x}\left(t+1\right)\text{x}{\left(t\right)}^{T}-\beta^{-\frac{1}{2}}\text{x}\left(t\right)\text{x}{\left(t+1\right)}^{T}\right) \end{array}$$

At each step, the excitatory recurrent connections are scaled by synaptic normalization (with *m*∈{1, 2}):

(18) $$\begin{array}{l} {\text{W}}_{m,ij}^{EE}\leftarrow {\text{W}}_{m,ij}^{EE}\left(1+\eta_{sn}\left(\frac{1}{\underset{k}{\sum }{\text{W}}_{1,ik}^{EE}+\underset{k}{\sum }{\text{W}}_{2,ik}^{EE}}\right)-1\right) \end{array}$$

Here, η_*sn*_ is usually set to 1 unless stated otherwise, i.e., synaptic normalization acts instantaneously. Please note that since both matrices are scaled by their combined total weight, the total maximal activation is still 1, as in the original model. The other connection matrices are initially also scaled to a maximal activation of 1.

To model synaptic failure, each excitatory-excitatory synapse is set to fail with the probability *p*_fail_ at each time step. This is included in both the update rules and the STDP rules by ignoring STDP events for the synapses that failed to contribute to the updated activity.

The three remaining plasticity mechanisms are left unchanged:

The inhibitory spike-timing-dependent plasticity (iSTDP) acting on the connections from the inhibitory to the excitatory connections in the following way:

(19) $$\begin{array}{l} \Delta {\text{W}}_{ij}^{EI}\left(t\right)=-\eta_{inhib}{\text{y}}_{i}\left(t-1\right)\left(1-{\text{x}}_{i}\left(t\right)\left(1+1/\mu_{IP}\right)\right) \end{array}$$

This rule aims to balance the excitatory and inhibitory drive to excitatory neurons by reducing the connection from an inhibitory to an excitatory neuron by η_*inhib*_ whenever a spike in the inhibitory neuron isn't followed by an excitatory spike and increasing the connection by η_*inhib*_∕μ_*IP*_ when an inhibitory spike is followed by an excitatory spike.

The structural plasticity acts on both excitatory recurrent matrices independently by randomly adding a connection with a probability of *p*_*struct*_ = 0.1 and an initial weight of *w*_*sp*_ = 0.001 at every time step.

The intrinsic plasticity adapts the excitatory thresholds the following way:

(20) $$\begin{array}{l} {\text{T}}^{E}\left(t+1\right)={\text{T}}^{E}\left(t\right)+\eta_{\text{IP}}\left(\text{x}\left(t\right)-H_{\text{IP}}\right) \end{array}$$

This ensures that the excitatory units ***x*** fire with a mean rate of *H*_*IP*_ = 0.1 spikes per second. η_*IP*_ is set to 0.01 as in the original paper.

## Author contributions

CH, DM, JT conceived and designed the work. JT derived Model 1. CH did the simulations and analysis for Model 1 and Model 3. DM did the simulations and analysis for Model 2. CH, DM, JT wrote the manuscript.

## Funding

All authors were funded by the Quandt foundation.

### Conflict of interest statement

The authors declare that the research was conducted in the absence of any commercial or financial relationships that could be construed as a potential conflict of interest.

## Supplementary material

Figure S1 **Distributions of η(*t*) for the *detailed balance* and *global balance* simulation. (A)** Experimentally measured distribution of η(*t*) for the *detailed balance* simulation. η(*t*) was computed at each step as $-\frac{\overset{N}{\underset{i=0}{\sum }}P_{i}^{1}\left(t\right)+P_{i}^{2}\left(t\right)}{\overset{N}{\underset{i=0}{\sum }}X_{i}^{1}\left(t\right)+X_{i}^{2}\left(t\right)}$. **(B)** For the *global balance* simulation, η(*t*) is drawn from a similar normal distribution: η(*t*) ~ *normal*(μ = −0.002, σ = 0.001).

Figure S2 **Single neuron simulation with reduced synaptic normalization timescale**. Behavior is demonstrated for reduced normalization timescales in the case of η_*SN*_ = 0.1 and η_*SN*_ = 0.001, in terms of mean alignment **(A)** and mean CV **(B)** between parallel synapses. Shading shows the standard error of the mean over 1000 weight pairs.

Figure S3 **Single neuron simulation with multiplicative STDP**. Multiplicative STDP with soft bounds is implemented by multiplying the weight change by a factor of (1−*w*∕*w*_*max*_). Behavior is demonstrated in terms of mean alignment **(A)** and mean CV **(B)** between parallel synapses. Shading shows the standard error of the mean over 1000 weight pairs.

Figure S4 **Single neuron simulation with alternate plasticity mechanisms (weight-dependent synaptic failure and subtractive normalization)**. Behavior is shown under LTP-biased STDP (depression 0.75 × as strong as potentiation). For weight-dependent failure, failure rate is calculated as $p\left(w\right)=1-e^{\lambda \left(w+w_{0}\right)}$. We try two different parameter sets. For weight dependent failure a, λ = (log(0.2) − log(0.8))∕(2.5 *mV*) and *w*_0_ = 2.5 log(0.8) *mV*∕(log(0.2) − log(0.8)) (calculated to yield a median release rate of 0.8 and a minimum release rate of 0.2 for the initial weight distribution, and leading to a mean release rate of 0.68 with a variance of 0.07 on the final weight distribution), and for weight dependent failure b, λ = (log(0.1) − log(0.5))∕(2.5 *mV*) and *w*_0_ = 2.5 log(0.5) *mV*∕(log(0.1) − log(0.5)) (calculated to yield a median release rate of 0.9 and a minimum release rate of 0.5 for the initial weight distribution, and leading to a mean release rate of 0.83 with a variance of 0.02 on the final weight distribution). Subtractive normalization is implemented by, at each execution of the process, subtracting from each weight the difference in the target normalization value and the sum of the weights divided by the number of synapses, clipping at zero if necessary. Alignment is demonstrated to be present but weakened for weight-dependent failure and sensitive to the failure function, and minimal for subtractive normalization. Alignment is shown in terms of mean alignment **(A)** and mean CV **(B)** between parallel synapses. Shading shows the standard error of the mean over 1000 weight pairs.

Figure S5 **Key effects in network model hold for slow synaptic normalization**. We simulated the SORN model with the normalization rate set to 0.1 instead of 1, no LTP bias, and 20% failure rate. **(A)** Parallel synapses align. The pair with the maximal final mean weight (blue and dashed) and a randomly selected pair (green and dotted) are plotted for the beginning of the simulation. **(B)** The CV drops below the experimentally reported values for different failure probabilities. **(C)** STDP dynamics at the beginning of the simulation. The total weight change (green) is biased toward LTP. **(D)** Mean synapse size and their CV are significantly correlated. Shaded areas are standard deviations over five independent simulations.

Figure S6 **Key effects in network model hold for weight-dependent synaptic failure**. We simulated the SORN model with weight-dependent synaptic failure *p*(*fail*|*w*) = *e*^−6*(*w*+0.1)^. The parameters of the exponential were set to achieve both an average failure probability similar to the ones tested so far in order to allow comparison and a high dynamic range. **(A)** The final weights after simulating the SORN without biased LTP (blue dots) can be fitted by a log-normal distribution (green line). The exponential weight-dependent failure probability (red line and axis) looks sigmoidal due to the log-scale on the x-axis. **(B)** The average failure probability changes over time in line with the connection fraction: a smaller connection fraction implies less but stronger weights and thereby a lower average failure probability. The final probability of failure is around 0.3 in line with our previous simulations. **(C)** The development of the coefficient of variation is similar to the simulations with weight-independent synaptic failure. **(D)** The qualitative development of the connection fraction is similar to the simulations with weight-independent synaptic failure. The final connection fraction is lower. Shaded areas are standard deviations over five independent simulations.
