# Supplementary figures and images for: Precise Synaptic Efficacy Alignment Suggests Potentiation Dominated Learning
Source: Front Neural Circuits. 2016 Jan 13;9:90. doi: 10.3389/fncir.2015.00090 (PMC4711154; doi:10.3389/fncir.2015.00090)

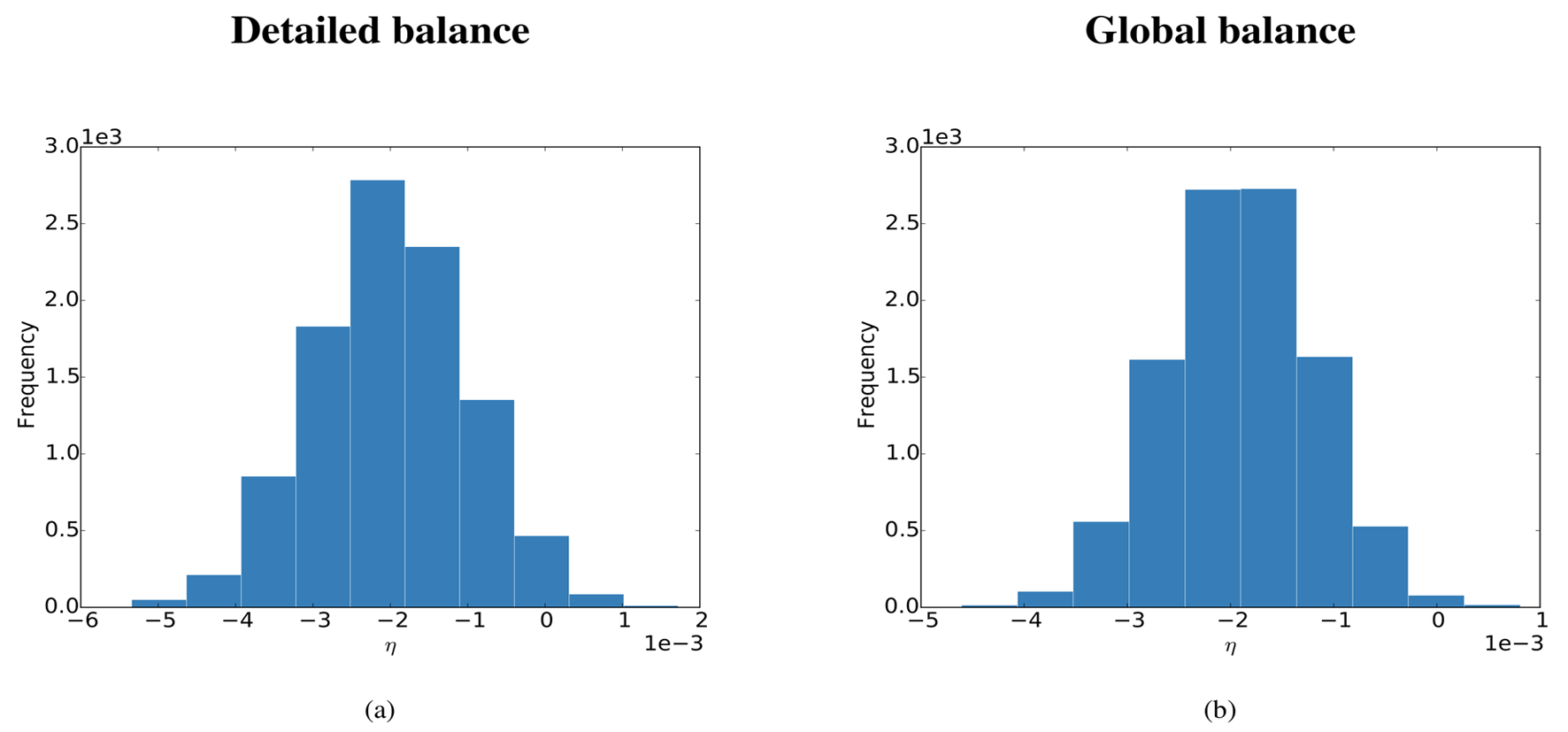

Supplement: Figure S1 — Distributions of η(t) for the detailed balance and global balance simulation. (A) Experimentally measured distribution of η(t) for the detailed balance simulation. η(t) was computed at each step as -∑i=0NPi1(t)+Pi2(t)∑i=0NXi1(t)+Xi2(t). (B) For the global balance simulation, η(t) is drawn from a similar normal distribution: η(t) ~ normal(μ = −0.002, σ = 0.001). [file Image1.TIFF]

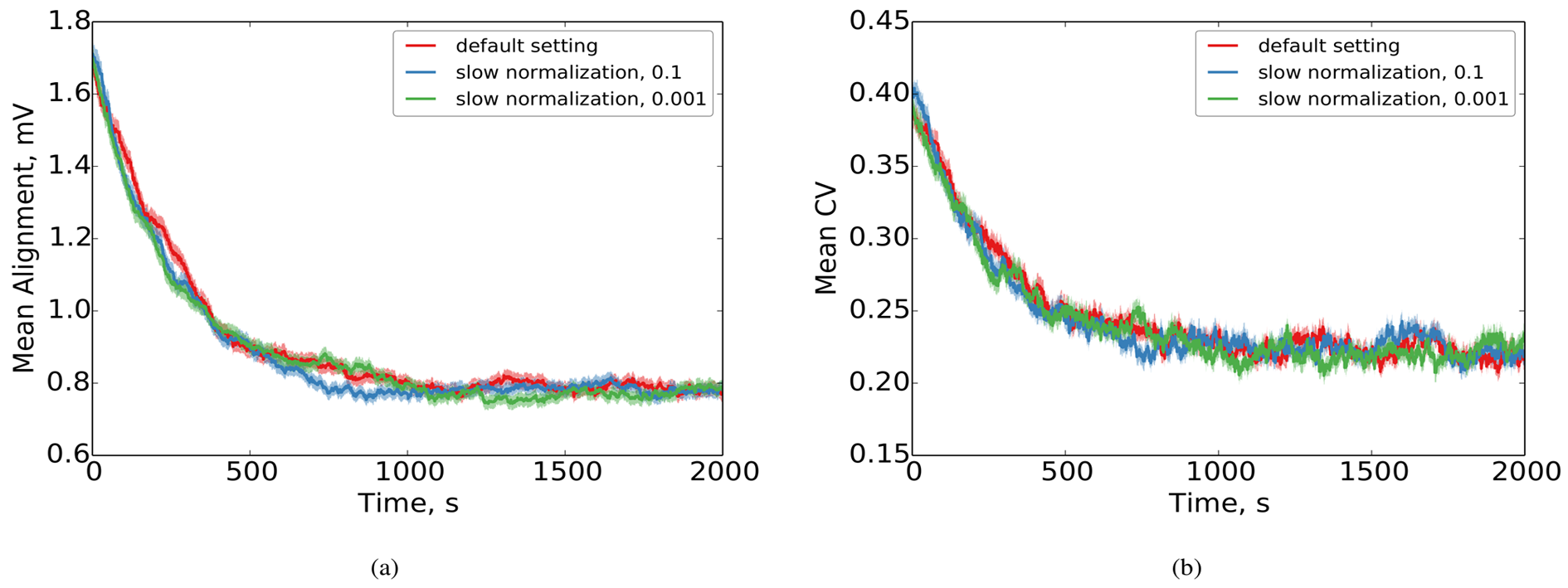

Supplement: Figure S2 — Single neuron simulation with reduced synaptic normalization timescale. Behavior is demonstrated for reduced normalization timescales in the case of ηSN = 0.1 and ηSN = 0.001, in terms of mean alignment (A) and mean CV (B) between parallel synapses. Shading shows the standard error of the mean over 1000 weight pairs. [file Image2.TIFF]

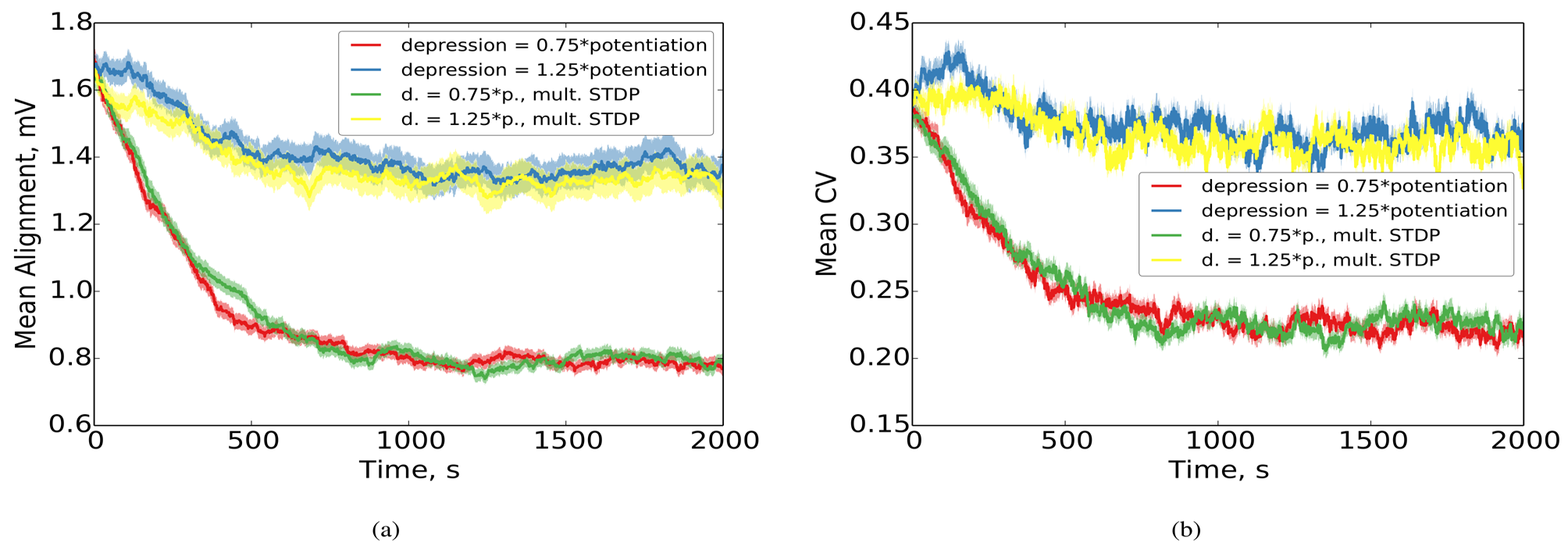

Supplement: Figure S3 — Single neuron simulation with multiplicative STDP. Multiplicative STDP with soft bounds is implemented by multiplying the weight change by a factor of (1−w∕wmax). Behavior is demonstrated in terms of mean alignment (A) and mean CV (B) between parallel synapses. Shading shows the standard error of the mean over 1000 weight pairs. [file Image3.TIFF]

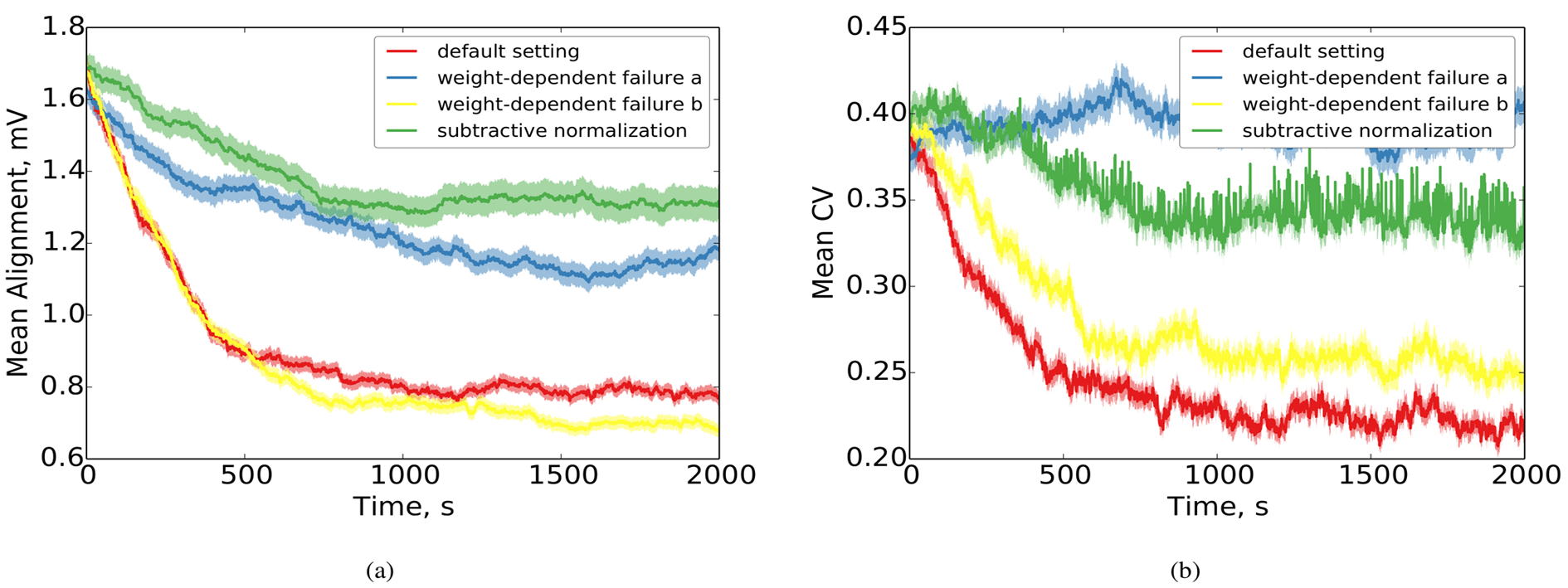

Supplement: Figure S4 — Single neuron simulation with alternate plasticity mechanisms (weight-dependent synaptic failure and subtractive normalization). Behavior is shown under LTP-biased STDP (depression 0.75 × as strong as potentiation). For weight-dependent failure, failure rate is calculated as p(w)=1-eλ(w+w0). We try two different parameter sets. For weight dependent failure a, λ = (log(0.2) − log(0.8))∕(2.5 mV) and w0 = 2.5 log(0.8) mV∕(log(0.2) − log(0.8)) (calculated to yield a median release rate of 0.8 and a minimum release rate of 0.2 for the initial weight distribution, and leading to a mean release rate of 0.68 with a variance of 0.07 on the final weight distribution), and for weight dependent failure b, λ = (log(0.1) − log(0.5))∕(2.5 mV) and w0 = 2.5 log(0.5) mV∕(log(0.1) − log(0.5)) (calculated to yield a median release rate of 0.9 and a minimum release rate of 0.5 for the initial weight distribution, and leading to a mean release rate of 0.83 with a variance of 0.02 on the final weight distribution). Subtractive normalization is implemented by, at each execution of the process, subtracting from each weight the difference in the target normalization value and the sum of the weights divided by the number of synapses, clipping at zero if necessary. Alignment is demonstrated to be present but weakened for weight-dependent failure and sensitive to the failure function, and minimal for subtractive normalization. Alignment is shown in terms of mean alignment (A) and mean CV (B) between parallel synapses. Shading shows the standard error of the mean over 1000 weight pairs. [file Image4.TIFF]

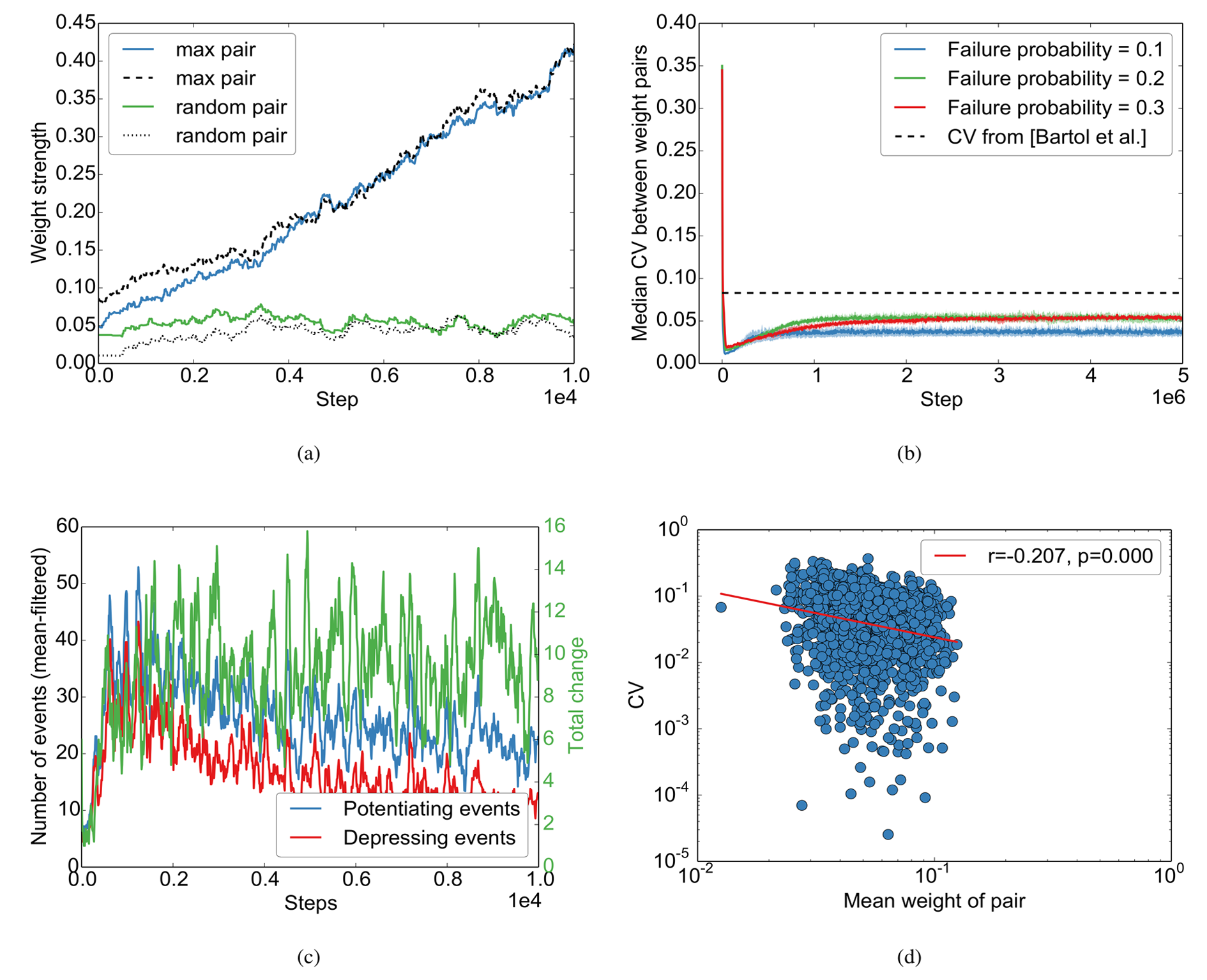

Supplement: Figure S5 — Key effects in network model hold for slow synaptic normalization. We simulated the SORN model with the normalization rate set to 0.1 instead of 1, no LTP bias, and 20% failure rate. (A) Parallel synapses align. The pair with the maximal final mean weight (blue and dashed) and a randomly selected pair (green and dotted) are plotted for the beginning of the simulation. (B) The CV drops below the experimentally reported values for different failure probabilities. (C) STDP dynamics at the beginning of the simulation. The total weight change (green) is biased toward LTP. (D) Mean synapse size and their CV are significantly correlated. Shaded areas are standard deviations over five independent simulations. [file Image5.TIFF]

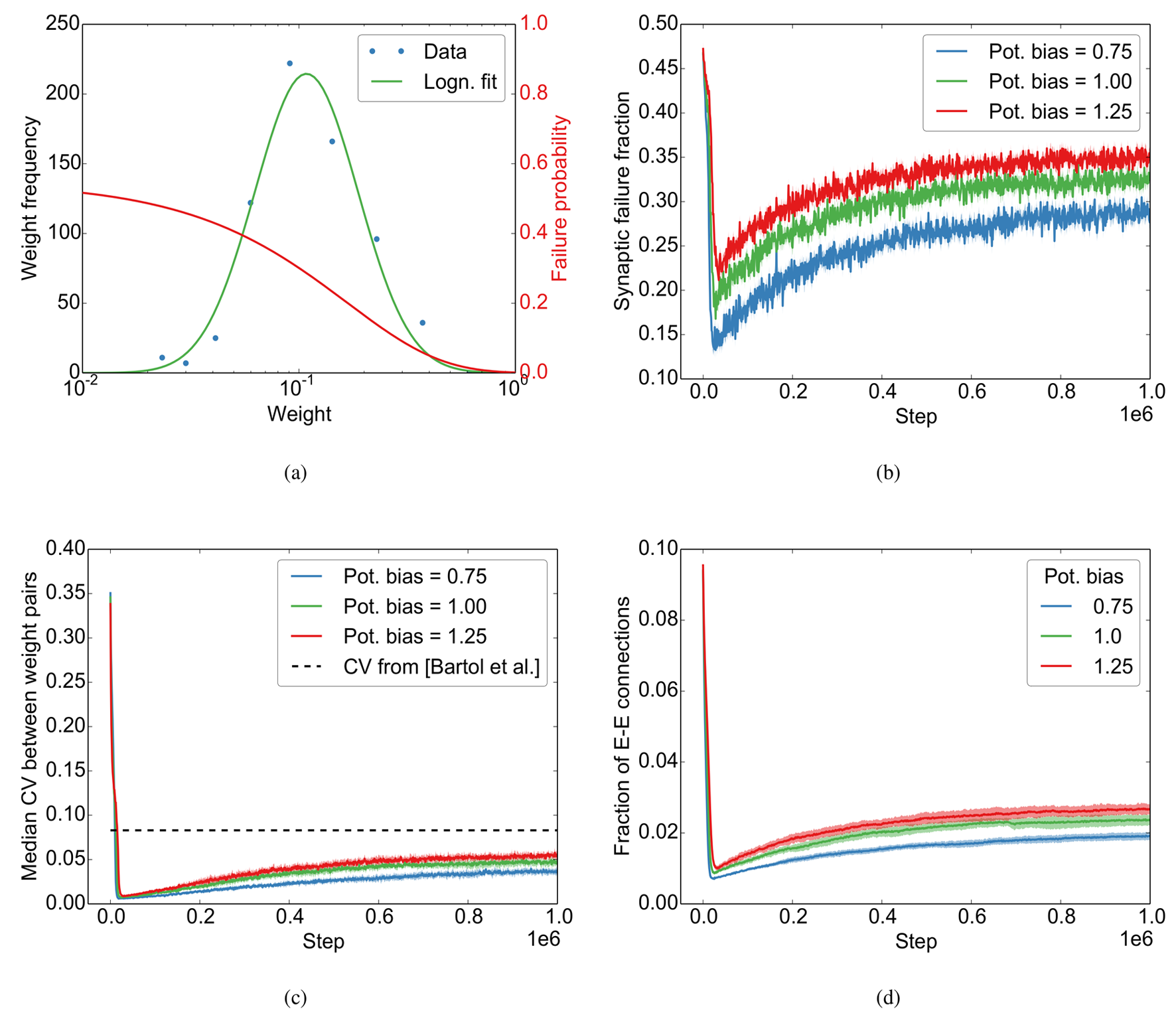

Supplement: Figure S6 — Key effects in network model hold for weight-dependent synaptic failure. We simulated the SORN model with weight-dependent synaptic failure p(fail|w) = e−6*(w+0.1). The parameters of the exponential were set to achieve both an average failure probability similar to the ones tested so far in order to allow comparison and a high dynamic range. (A) The final weights after simulating the SORN without biased LTP (blue dots) can be fitted by a log-normal distribution (green line). The exponential weight-dependent failure probability (red line and axis) looks sigmoidal due to the log-scale on the x-axis. (B) The average failure probability changes over time in line with the connection fraction: a smaller connection fraction implies less but stronger weights and thereby a lower average failure probability. The final probability of failure is around 0.3 in line with our previous simulations. (C) The development of the coefficient of variation is similar to the simulations with weight-independent synaptic failure. (D) The qualitative development of the connection fraction is similar to the simulations with weight-independent synaptic failure. The final connection fraction is lower. Shaded areas are standard deviations over five independent simulations. [file Image6.TIFF]
